# Merkel cell carcinoma in a malignant pleural effusion: case report

**DOI:** 10.1186/1742-6413-1-5

**Published:** 2004-11-18

**Authors:** Misty M Payne, Anne E Rader, Denis M McCarthy, William H Rodgers

**Affiliations:** 1 Oregon Health & Science University 3181 SW Sam Jackson Park Road, L471 Portland, OR 97239 USA

## Abstract

**Background:**

Merkel cell (neuroendocrine) carcinoma is a small round blue cell malignant neoplasm that primarily presents in the skin. The diagnosis of Merkel cell carcinoma in a pleural fluid is challenging because of the morphological similarity to many other malignant neoplasms. Immunohistochemical stains can be essential to establish the diagnosis of Merkel cell carcinoma.

**Case presentation:**

A 77 year-old woman presented with a mass in her right buttock thought clinically to be a boil or sebaceous cyst. Upon histopathologic review including immunohistochemical analysis, a diagnosis of Merkel cell carcinoma was rendered. Wide-excision and sentinel lymph node biopsy revealed negative margins and no evidence of metastasis. Ten months later she complained of bone pain and a bone scan revealed multiple lesions. An abdominal CT scan revealed a T4 vertebral mass and local radiotherapy was administered. Two months later the patient presented with shortness of breath. A chest radiograph showed an effusion and thoracentesis was performed. The fluid was confirmed to contain metastatic Merkel cell carcinoma by cytology and immunohistochemical analysis.

**Conclusions:**

Merkel cell carcinoma is an aggressive neoplasm that can, despite careful surgical management, occasionally present as a malignant pleural effusion in a relatively short time period. Immunohistochemical analysis can aid in confirming this rare outcome.

## Background

The Merkel cell is the namesake of an F. Merkel who in 1875 described distinct round cells in the basal layer of the epidermis [1]. The distinctive Merkel cell with its round shape and salt and pepper chromatin is known to be present in the epidermis and dermis [2]. Toker first described Merkel cell carcinoma in 1972 and along with Tang concluded the cells of origin to be derived from the neural crest [3,4]. More recently, Sibley discussed the idea that the cells may be derived from the pleuripotential basal cells of the epidermis that surround hair follicles and function as touch receptors in the skin [5]. Merkel cell carcinoma is a tumor of the dermis and can easily be mistaken for other malignant small round blue cell tumors such as lymphoma, amelanotic melanoma, and metastatic small cell carcinoma [6]. Historically, electron microscopy demonstrating cytoplasmic microfilaments and neurosecretory granules was the key to diagnosing Merkel cell carcinoma [7]. Immunohistochemical approaches are now used to confirm a diagnosis of Merkel cell carcinoma.

## Case Report

A 77 year old female presented to her primary care physician with a complaint of a right buttock mass. Her past medical history was significant for colonic adenocarcinoma treated surgically, twelve years prior. The skin overlying the 2 × 3 cm firm, non-fluctuant, non-tender mass was erythematous. Initially the mass was thought to be a boil or carbuncle. Six weeks later during a visit with a surgical oncologist for routine follow-up of her colonic adenocarcinoma resected 13 years prior, the patient noted that the mass was becoming quite painful and enlarging rapidly. The patient was then taken to the OR for local excision. Pathology revealed a small round blue cell tumor forming large nests and infiltrating as single cells between thick strands of fibrous tissue in the dermis and extending to the subcutaneous fat. There were abundant mitoses, a lack of necrosis or obvious vascular invasion. The epidermis was uninvolved. The histologic and immunochemical findings supported the diagnosis of Merkel cell carcinoma. A wide local excision and sentinel lymph node biopsy were performed. One sentinel lymph node was negative for malignancy and there was no residual tumor identified in the excision. No adjuvant therapy was given.

Ten months after the initial presentation the patient had increasing shoulder pain that radiated into the chest and flank. A CT of the thoracic spine revealed a 3.0 cm mass in the area of the fourth thoracic (T4) vertebra. A CT guided needle biopsy of the area revealed metastatic Merkel cell carcinoma. A bone scan revealed additional areas of concern in the right humerus and bilateral distal femurs. The patient received radiation therapy to the thoracic spine and right distal femur for palliation of bone pain.

**Figure 1:**
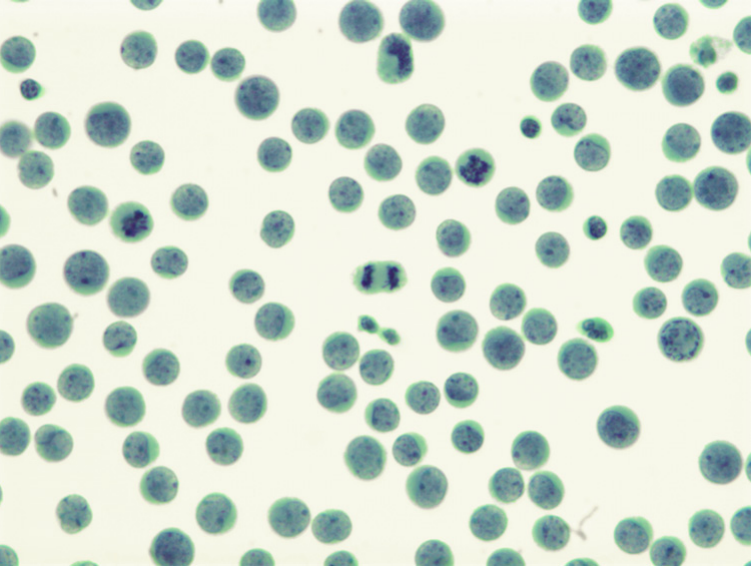
Pleural fluid at 630x demonstrating small round cells with salt and pepper chromatin. Note multiple mitotic figures

**Figure 2:**
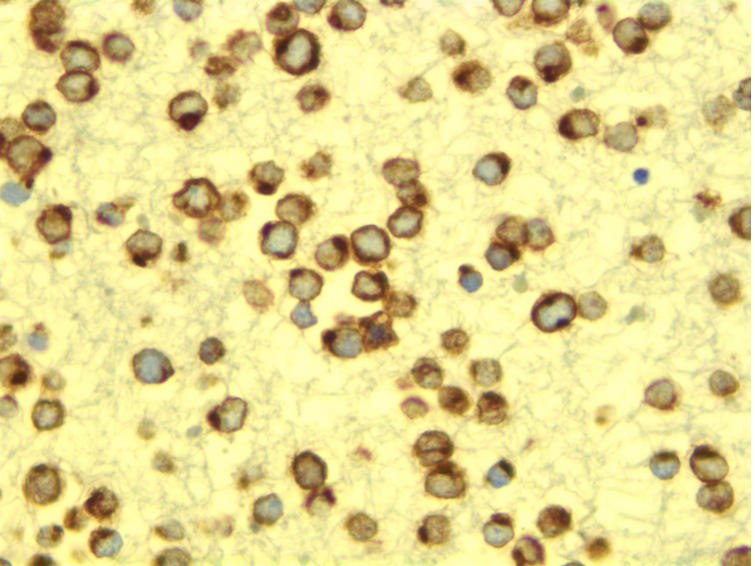
Cell block stained with CK20 demonstrating sharp perinuclear dot like pattern.

A year after first presenting for a right buttock mass, the patient was admitted to the hospital for increasing dyspnea. A chest radiograph revealed a large right pleural effusion, which was drained for both therapeutic and diagnostic purposes. The fluid was grossly bloody and cytology revealed abundant, round, basophilic single cells, many in mitosis, with characteristic granular, "salt and pepper" nuclear chromatin (Figure 1). A cellblock was made and immunohistochemical staining with CK20 revealed characteristic positivity in a dot like rim pattern (Figures 2).

## Conclusions

The diagnosis of Merkel cell carcinoma in pleural fluid is challenging partially because of its rarity. To our knowledge this is the first case report of Merkel cell carcinoma in a pleural fluid characterized with immunohistochemical stains. Wason and Friedman described the only other reported case of pleural effusion due to metastatic Merkel cell carcinoma in 1985 [7]. In that report, the diagnosis was confirmed by electron microscopy that demonstrated cytoplasmic microfilaments and numerous dense-core, peripheral, neurosecretory granules. The light microscopic morphologic characteristics of Merkel cells in pleural fluid yields a large list of possible diagnosis including small cell lung carcinoma, carcinoid tumor, lymphoma, plasmacytoma, pancreatic carcinoma small cell type, and peripheral neuroectodermal tumor (PNET).

Since Merkel cell carcinoma rarely appears in pleural fluid and small cell lung carcinoma frequently does the pathologist must have the salient cytomorphologic features of Merkel cell carcinoma in mind to make the diagnosis in the absence of clinical history. The most characteristic features of Merkel cell carcinoma in pleural fluid include convolution of nuclei and lack of prominent nuclear molding. Once Merkel cell carcinoma has entered the differential diagnosis the case can be pursued.

With immunohistochemistry, it is now possible to definitively diagnose Merkel cell carcinoma in pleural fluid without electron microscopy. The immunohistochemical profile of Merkel cell carcinoma is distinct and has been well established on paraffin embedded tissue [8]. Tumor cells consistently express low molecular weight keratins in the form of a perinuclear dot like pattern typical of neuroendocrine carcinomas. Merkel cell carcinomas also label for neuron specific enolase (virtually all), chromogranin B (100%), chromogranin A (72%), secretoneurin (22%), and synaptophysin (39%) [5,9]. Among neuroendocrine carcinomas, CK20 positivity in a dot like rim pattern is seen only in Merkel cell and salivary gland small cell carcinomas [10] Other small cell tumors that are CK20 positive include bronchogenic small cell carcinoma (0.03%), small cell cervical carcinomas (9%), and small cell carcinomas of salivary glands (60%) but not in the characteristic dot-like pattern [11]. Neuron specific enolase is positive in almost all Merkel cell carcinomas [5]. Carcinoid tumors stain for synaptophysin and chromogranin, but not CK20. Reactivity with CD45 is diagnostic of a hematopoietic lineage; in addition, apart from vimentin, hematologic malignancies do not generally react with antibodies to other lineage specific markers. Plasmacytomas express CD138 and CK8 [10]. CD 99 positivity suggests PNET; however, one series found CD99 to be positive in 40% of Merkel cell tumors [12].

Metastatic Merkel cell carcinoma as the cause of a malignant pleural effusion is a rare occurrence. However, such an aggressive neoplasm as Merkel cell, which is often widely metastatic, may be a more common cause of malignant pleural effusion than recognized. Morphologically, the tumor cells in a body fluid could easily be mistaken for a malignancy of another origin. In the work-up of the pleural effusion caused by a small round blue cell process, inclusion of immunohistochemical markers specific to Merkel cell carcinoma may be prudent.

## Competing interests

The authors declare that they have no competing interests.

## Authors' contributions
